# The Impact of the Media Portrayal of Coeliac Disease: A Qualitative Study

**DOI:** 10.7759/cureus.32444

**Published:** 2022-12-12

**Authors:** Satvik R Verma, Manpreet Bains

**Affiliations:** 1 Trauma and Orthopaedics, Kingston Hospital, London, GBR; 2 Faculty of Medicine & Health Sciences, University of Nottingham, Nottingham, GBR

**Keywords:** culture and media, gluten-free, gluten-free diet, celiac disease (ced), coeliac

## Abstract

Background: Coeliac disease (CD) is a topic that has gained momentum in recent years due to an increase in the rates of diagnosis and a rise in the number of people following a gluten-free diet (GFD). Previous studies have shown the ability of the media to influence the behavior of the public, particularly in relation to healthcare. The media portrayal of CD, and its significance, has not yet been explored.

Objectives: This study aims to describe and analyze the nature of the media coverage of CD. The implications of the nature of messages should be considered, and any temporal change in content explored.

Methods: A document analysis of local and national UK newspaper articles over three weeks, from May 2^nd^ to May 22^nd^, 2016, was conducted, ensuring coverage of articles from Coeliac Awareness Week (commencing May 9^th^). Articles containing instances of CD-related language were collected by Kantar Media ([London, UK] a media intelligence company) for their client - Coeliac UK (Buckinghamshire, UK), and analyzed using a combination of thematic and content analysis techniques. An inductive approach was used to code articles into themes, with frequency data also presented.

Results: Four hundred eighty-eight articles were included in the analysis, with 233, 117, and 138 articles in weeks one, two, and three, respectively. Six overarching themes were identified: events around Awareness Week and food content noted as gluten-free (GF), as well as raising awareness, encouraging people to seek help, and other health implications and perceptions of CD and the GFD, of which a significant proportion consisted of articles by Coeliac UK. The increasing popularity of the GFD emerged as a theme, encompassing the growth of the GF industry and celebrity endorsements. Notably, positively and negatively framed articles were identified, with most instances of negative articles occurring in week three.

Conclusions: Coeliac Awareness Week was found to have had an influence on the content of newspaper articles, given the increase in the number of negative articles in week three and the significantly higher number of articles in week one. This mixed messaging was considered to negatively impact the potential and current patients with CD, especially in relation to GFD adherence and diagnosis rates.

## Introduction

Coeliac disease (CD) is a chronic autoimmune enteropathy precipitated by genetic and environmental factors, namely exposure to dietary gluten [1]. Clinical presentation of CD varies and can differ depending on age [2]. Classical symptoms include gastrointestinal problems such as diarrhea, steatorrhea, and abdominal distention, as well as failure to thrive. Generally, symptoms present at around 6-18 months; however, in recent years, patients have been presenting with symptoms at a later age and without classical symptoms [3]. CD has become increasingly prevalent in western society over the past few decades (currently around 1% of the population), with a significant proportion still undiagnosed [3]. Diagnosis of CD relies on clinical, serological, and histological evidence, and the increase in rates of diagnosis can be partly attributed to the use of sensitive serology testing. Confirmation of CD requires an endoscopic small intestinal biopsy, which is the gold standard for diagnosis [4].

A gluten-free diet (GFD) is the recommended treatment for CD. Untreated CD can lead to complications that include premature/early mortality, poor growth, and osteoporosis; however, the majority of these complications can be avoided by following a GFD [5,6]. Life-long adherence to a GFD can be complex and costly and will require considerable changes in eating habits that can be challenging for an individual. There are significant challenges associated with adherence to a GFD, including cost, availability of GF products, and psychological barriers. Such barriers, especially restrictions in social situations, can lead to dietary non-adherence as a result of a poorer quality of life [7,8]. The rate of GFD adherence is unclear, with estimates ranging from 45%-80% [6,9].

The steady growth of the gluten-free (GF) market and the projection of growth in the future mirrors the increasing incidence of CD in the population. However, much of this trend can also be attributed to those who do not have CD or a wheat allergy but simply feel better and ‘healthier’ when avoiding gluten [10]. The media portrayal of gluten and gluten-related disorders has caused many individuals to denounce and exclude gluten from their diet in the absence of any reasonable medical reasons. This growing interest in the GFD could be a result of increased availability of GF products, self-diagnosis of gluten sensitivity by individuals, and the perception that the diet is ‘healthier’, fueled by media representation. This trend can lead to a diagnostic delay in those who do have CD, as well as those without, as it can ‘muddy the waters’ in the eyes of the clinician.

Coeliac UK is a charity for people with CDs. The organization supports individuals with CD and aims to improve their experience of healthcare, with campaigning for greater awareness of CD being a principal objective. Their ‘Is it coeliac disease?’ campaign, launched in 2015, aims to halve the length of time for a diagnosis (currently 13 years) and reduce misdiagnosis of irritable bowel syndrome (IBS) by 50% [11]. Awareness Week, which took place from 9-15 May 2016, is a significant part of the campaign and consisted of a social media takeover, spreading information through the media, pop-up events in areas where rates of diagnosis are lower, and National Coeliac UK Leafleting Day.

The increasing incidence of CD, a focus on increasing the number of diagnoses by Coeliac UK, and the lack of knowledge about CD amongst individuals as well as the medical community gives scope for a study to investigate the portrayal of CD in the media. The media, especially traditional newspapers, have a relatively broad reach and so play an important role in promoting awareness in society. Studying the nature of the messaging around CD during Coeliac Awareness Week, with a before and after period is important. This would be beneficial to public health as although there have been many studies outlining the impact of the media, and especially mass media campaigns, on other health issues such as tobacco and alcohol use, literature regarding CD is very limited. Thus, it would be worth considering whether findings from other areas (e.g., around framing) are transferable to a topic that is gaining momentum and popularity in society. This study would be one of the first to analyze the representation of CD in the media and the portrayal of the issues surrounding the growing trend of GFDs. The qualitative nature of this study is important to engage a dialectic process between the questions being asked and the data collected. An inductive thematic analysis was undertaken due to the nature of the available data and the lack of previous research. This study could therefore provide the initial framework and generate a hypothesis that can be useful in further research regarding CD and the media and provide a framework for quantitative research.

## Materials and methods

A document analysis of newspaper articles was conducted to explore the coverage of CD. A combination of thematic and content analysis techniques was utilized. In relation to thematic analysis, an inductive approach was used to generate codes, following the framework outlined by Braun and Clarke [12]. Coeliac UK commissioned Kantar Media to collect media coverage of the CD. Kantar Media is a leading media intelligence company specializing in broadcast media. Kantar Media was instructed by Coeliac UK to collect articles that include any of the following search terms: ‘gluten-free’, ‘coeliac disease’, ‘gluten intolerance’, and ‘Coeliac UK’. Identified articles are sent to Coeliac UK, which organizes articles according to the date of publication. Coeliac UK provided articles from January to June 2016. 

Due to the large number of newspaper articles collected by Kantar Media over the six months, the decision was made to focus on data from three weeks: May 2^nd^ to May 22^nd^, 2016. This time frame was selected to ensure coverage of articles from Coeliac Awareness Week (May 9^th^ to 15^th^), which is a key event in the calendar for Coeliac UK, and coverage of the disease in the media and the week before and following. This time frame was chosen not only to make the data more manageable but because it would help to explore whether media coverage (as well as the framing of the articles/condition) and the nature of this coverage change. Articles from national and local newspapers in print and online versions, as well as newspaper supplements and features, were included in the analysis. Several articles that included one or more of the search terms were found in magazines, specialized journals, and blog posts. These were excluded from the analysis because the focus of this study was on newspaper articles that were considered to have the most reach in terms of the number of readers. Hence, only newspaper articles and related features, along with their online versions were included.

Data were analyzed by combining content and thematic analysis techniques. First, themes were identified inductively based on frameworks derived from Braun and Clarke [12]. Second, a content analysis was conducted in which frequency data is presented. The inductive approach that was used for thematic analysis meant that conceptualization of the meaning of the derived code did not take place until the complete dataset was analyzed to ensure the characteristics of data-driven code over theory-driven code. This inductive approach was used, given the paucity of previous research in this topic area. 

The first five days of articles were initially read and re-read to gain familiarity with the source material. The raw material was then summarized in detail and noted in a Microsoft Excel file (Washington, USA) that enabled the development of initial ideas and codes. This allowed for an in-depth review of the data, as well as for the raw information to be condensed, establishing increasing familiarity with the data. Similar patterns and characteristics were identified amongst the sub-samples to develop preliminary codes. From this, preliminary themes were generated. A theme is described by Boyatzis as ‘a pattern in the information that, at minimum, describes and organizes the possible observations and, at maximum, interprets aspects of the phenomenon’ [13].

Two days from each week with the highest number of publications were selected for independent double coding by the project supervisors, giving six days’ worth of data in total. This resulted in 39% of data being double coded to ensure validity of the codes through investigator triangulation. Preliminary themes were considered and discussed between the researcher and supervisors. Final themes and sub-themes were refined and defined, ensuring all aspects of the data relating to CD were covered appropriately.

## Results

**Figure 1:**
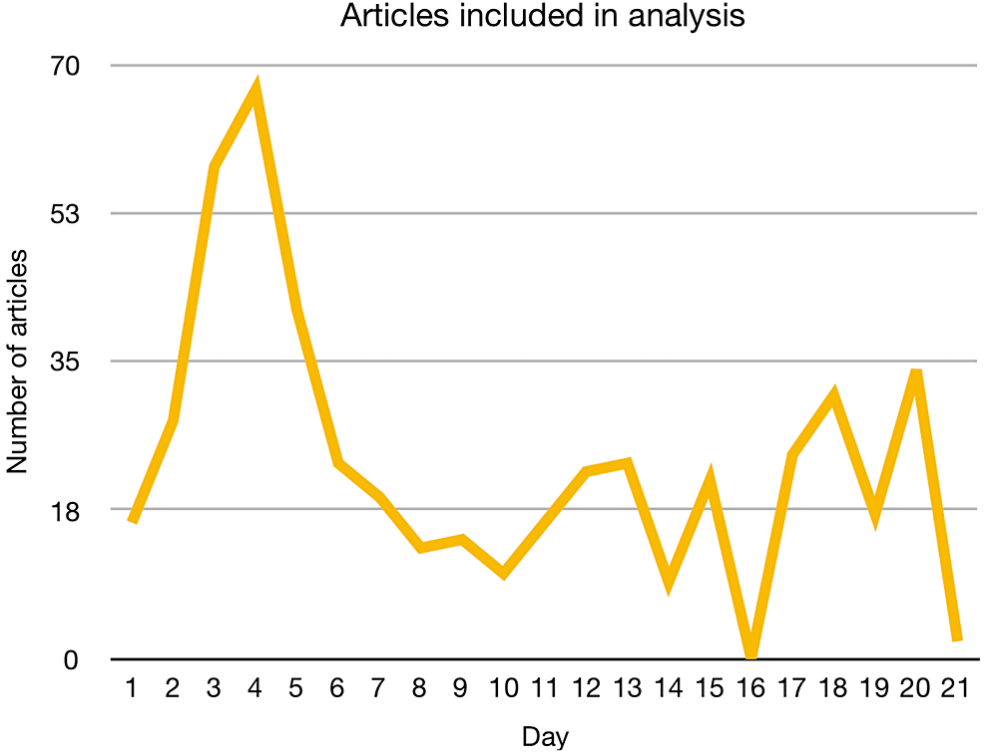
Number of articles per day

**Figure 2:**
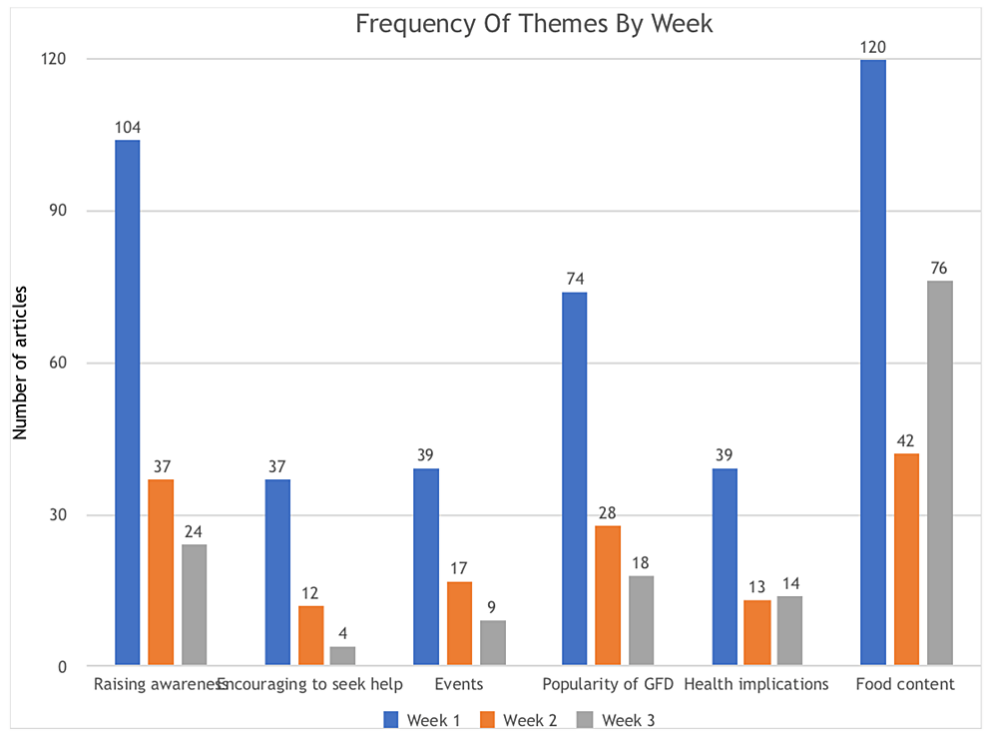
Frequency of themes by week GFD: Gluten-free diet

The three-week period from May 2^nd^ to May 22^nd^ 2016, yielded 984 articles, of which 488 were included in the analysis. Six major themes were identified: Raising awareness (n=163), encouraging people to seek help (n=50), events around Awareness Week (n=62), increasing popularity of the GFD (n=120), other health implications and perceptions of CD and the GFD (n=66) and food content noted as GF (n=238). This section covers the nature of the content according to each theme. The total number of articles can be seen in Figure 1, the frequency of each theme by week in Figure 2, and the ratio of each theme covered by local and national newspapers in Table 1.

**Table 1:** Ratio of local to national newspapers by theme GFD: Gluten-free diet, CD: Coeliac disease, GF: Gluten-free

Themes	Frequency in local newspapers	Frequency in national newspapers	Ratio
Raising awareness	150	15	10:1
Encouraging people to seek help	51	2	26:1
Increasing popularity of the GFD and associated problems	93	27	3:1
Other health implications and perceptions of CD and the GFD	46	20	2:1
Events around Awareness Week	58	4	15:1
Food content noted as GF	222	16	14:1

Raising awareness

One hundred sixty-three articles served to raise awareness around CD, both directly and indirectly. Articles by Sarah Sleet, Chief Executive of Coeliac UK, and Myles Fitt, Coeliac UK Head of Scotland, constituted many articles under this theme. These articles provided information on the nature of the disease and highlighted the symptoms commonly seen in CD, and urged the readers with any symptoms to take Coeliac UK’s online test. 

Following closer consideration, 98 articles, of which 39 were from Coeliac UK, described CD. The descriptions were consistent, where CD was commonly referred to as an autoimmune disease, with the pathophysiology outlined in simple terms - making it easy to understand by non-medical professionals. Most articles made a distinction between CD being a disease rather than an allergy; however, a minority of articles, such as those in The Plymouth Daily (8^th^ May, Web) and Portsmouth Today (5^th^ May, Web), referred to gluten as an ‘allergen’, implying that CD is an allergy. The prevalence, symptoms and management of CD were mentioned 99, 104 and 88 times, respectively, with 54 articles by Coeliac UK. Common symptoms described included osteoporosis, fertility problems and small bowel cancer, with prevalence usually given as 1%. 

Furthermore, the difficulties in diagnosing CD were outlined in 16 articles, helping to raise awareness of common symptoms. The length of time taken for diagnosis after the onset of symptoms was detailed by newspapers such as the Sunday Mail (9^th^ May, p12), which described the 11 years taken for a woman’s CD to be diagnosed, and The Selby Times, which reported the average of 13 years currently taken for confirmation of a CD diagnosis. Misdiagnosis of CD was a common issue, with patients misdiagnosed with conditions such as an underactive thyroid (The Fife Courier, 9^th^ May, p8), tonsillitis, and being ‘run down’ (Sunday Mail, 9^th^ May, p12), as well as IBS. The Guardian (9^th^ May, p4) claimed that a quarter of people with CD have been treated for IBS.

The impact of a CD diagnosis and a GFD on patients’ lives was discussed in 27 articles. Issues that arose included difficulties at meal times as well as the cost and quality of GF foods. An article by the Selby Times (5^th^ May, p20) discussed the limited options whilst eating out and fears surrounding the chef’s knowledge of CD - concerns that are reflected in other articles such as the Yorkshire Evening Post (11^th^ May, p15). Challenges surrounding the quality of GF products were discussed by chef Gearoid Lynch in an article by the Eire Daily Mirror (4th May, p26), who opined that GF food on the market is ‘unhealthy and pre-packaged’, with GF food described as ‘soulless’, ‘flavorless’ and ‘forlorn’ by The Stoke Sentinel (14^th^ May, p12). The withdrawal of GF food on prescription by the NHS was mentioned as an issue in three articles as ‘an area of concern’, especially for the most vulnerable, according to The Guardian (9^th^ May, p4).

Encouraging people to seek help

Fifty articles by Coeliac UK were identified, which encouraged people to seek help if they presented with common symptoms of CD, such as nausea, anemia, stomach cramps, vomiting, diarrhea, and constipation, amongst others. These articles went on to say: ‘If that is you, we encourage you to visit www.isitcoeliacdisease.org.uk and take Coeliac UK’s online assessment. The assessment provides you with results that you can take to your GP if your responses indicate a need for further tests.’ This online assessment allowed for the self-screening of patients, which may have allowed a greater number of people to seek help from their GP.

Events around Awareness Week

Sixty-two articles were identified that described events related to Awareness Week, such as charity events, book releases, and discounts on ‘free from’ ranges. Charity events included a sponsored trek (South Wales Echo, 6^th^ May, p27), sponsored bike ride (South Wales Echo, 10^th^ May, p23), and a variety concert (Edinburgh Evening News, 12^th^ May, p24). Book releases by chefs were relatively common, with articles that described Phil Vickery and his book as the most prominent. Numerous articles mentioned Awareness Week and marked it with a variety of activities, such as listing the best restaurants with GF menus (Coventry Telegraph, 12^th^ May, Web), giving talks in school (Barnsley Chronicle, 13^th^ May, p3), and serving GF baking (Stirling Observer, 6^th^ May, p28).

Increasing popularity of the GFD

An increased growth in the number of GF products suggested a growth in demand, along with the apparent popularity of following a GFD. This was mentioned in several articles, including The Sun, Daily Express, and Plymouth Herald. An article re-printed 43 times in various outlets mentioned Phil Vickery (Coeliac UK ambassador) detailing an increasing amount of GF foods being stocked in supermarkets, with popular brands such as Pizza Express, Young’s Fish Fingers, and Nestle Cereals more readily available in a GF form. Articles by the Daily Mail (5^th^ May, p48) advertising GF gelato formula from Iceland supermarket, Belfast Telegraph (12^th^ May), and The York Press (14^th^ May, p15) outlining eight new GF frozen products being stocked by Morrison’s supermarket support claim that the market is booming. Furthermore, the Mirror Online (5^th^ May, Web) reported a 70% increase in sales from Morrison’s ‘Free From’ range in the last year, including GF products, suggesting an increase in demand too. Interestingly, some of these articles alluded to the fact that a significant proportion of those that currently follow the GFD are those who do not follow it out of medical necessity. These include articles such as the Daily Mail (21^st^ May, p51) and The Daily Telegraph (21^st^ May, p11), which mentioned the rising rate of people with ‘food allergies [to gluten], even though only 1% of the population has coeliac disease’.

The role of celebrities (e.g., endorsements) was prominent in this theme. Articles showed celebrities from a variety of fields (chefs, athletes, and from the entertainment industry) endorsing GFDs. Phil Vickery, Gearoid Lynch (Eire Daily Mirror, 4^th^ May, p26), and Caroline Byron (The Ireland Times, 5^th^ May, p48) are chefs who were promoting GF food and recipes. Thomas Berdych, a top tennis player, said he can ‘sleep even better’ after cutting gluten from his diet (The Times, 21^st^ May, p11), and Kourtney Kardashian (reality TV star) mentioned that she ‘tries to stick to a mostly gluten-free diet’ (Mail Online UK, 2^nd^ May). However, an important distinction should be made between these occurrences. For instance, Phil Vickery, Thomas Berdych, and Gearoid Lynch promote following a GFD for medical reasons such as CD. Others endorse a GFD for reasons other than a medically diagnosed gluten-related disorder, such as Kourtney Kardashian and even the Catholic Church. An article by the Irish Mirror (8^th^ May, Web) described an increasing demand for GF communion wafers: ‘thanks in part to the current trend of gluten-free diets, priests across the country are making alternative communion wafers available.’

The increasing popularity of GF products has simultaneously given rise to new business products, which seem to make it easier to adhere to a GFD. Apps such as Shoptimix (Metro Scotland, 6^th^ May, p36) provides specially compiled shopping lists from nutritional experts with a GF option. Spoon Guru (Sunday Mirror, 8^th^ May, p4) ‘helps you make sense of the aisles and ensures you’re only putting the right stuff in your trolley,’ and FoodMaestro (Daily Express, 16^th^ May, Web) allows users to search and scan items to ensure they are safe to eat on a dietary restriction. Wiltshire Farm Foods (Nuneaton News, 18^th^ May, p25) have developed a home-delivery service for GF meals, whilst another company have developed ‘nutritious’ and ‘quick’ meals that are GF (Irish Times, 6^th^ May, Web).

Other health implications and perceptions of CD and the GFD

There were 66 articles that described the impact of CD or a GFD on other health related issues, which was a recurring theme throughout the data. Topics included encouraging women with CD to take a higher dose of folic acid supplement during pregnancy in newspapers such as The Daily Record (4^th^ May, p29) and Gloucestershire Echo (10^th^ May, p23). Fertility issues related to CD were mentioned in articles such as the Sunday Mail (8^th^ May, p12). Anemia was mentioned as a condition commonly associated with CD, and was acknowledged as a key symptom in identifying undiagnosed CD, along with the less common condition of osteoporosis in articles such as ‘The hidden disease that has no cure’ (Bradford Telegraph & Argus, 10^th^ May, p22) and by the Southern Daily Echo (10^th^ May, p19). However, no mention of anemia occurred in the week preceding Awareness Week, with all five instances occurring during Awareness Week. 

Several articles referred to an increased amount of fat in GF products compared to their gluten containing counterparts. The Mail Online UK’s article (16^th^ May, Web) claimed that “white bread from Asda contains 12.7g of fat - seven times the 1.9g of the ‘standard’ equivalent.” This statistic was reported by several other newspapers, such as the Daily Express (16^th^ May, Web) and Daily Telegraph (21^st^ May, p11), with large discrepancies in fat content also found in ready meals. Several articles reported the negative impact of following a GFD on the health of those who do not have a gluten-related disorder, such as The Sun Online (15^th^ May, p1), due to ‘potential nutritional deficiencies’ and ‘quality of life issues.’

These criticisms of GF products and the GFD were built upon by articles that expressed a negative attitude towards GFDs, which were thought of as a ‘fad’ and simply associated with ‘healthy eating’ or ‘eating clean’ e.g., The Sun (16^th^ May, p6), Daily Express (16^th^ May, p15) and The Sunday Telegraph (8^th^ May, p23). An article by The Times on the 2^nd^ of May described the GF lifestyle as ‘regimented’, ‘boring,’ and ‘square’, with the author of an article by the Daily Mail (21^st^ May, p51) saying that some of those who follow a GFD do it to ‘be special’, calling it the ‘bane of modern life’. This degree of skepticism towards the GFD was exemplified by a negative review of a GF television cooking show by the Norwich Evening News (13^th^ May, p8), ‘we were led to believe that whipping up an 18 ingredient Huevos Rancheros with chili, tomatoes, eggs, and guacamole served on a nest of spinach was a viable option for breakfast’. Furthermore, an article in the Sunday Telegraph (8^th^ May, p23) expressed concern that the term ‘clean eating’, which encompasses GFDs, could lead to eating disorders such as anorexia, and a Sunday Mail article (8^th^ May, p29) referred to the GFD as a ‘ludicrous lifestyle trend.’ Most articles that negatively portrayed GFDs were found to occur in the week after Awareness Week or on the last day of Awareness Week. Out of 21 articles identified, only six occurred in week one and the first six days of week two. Notably, a higher number of articles with negative framing were found in national newspapers (n=15) than in local newspapers (n=6) - a stark contrast considering the ratio of national and local newspapers in the analysis shown in Table 1.

Furthermore, the perception that the GFD was expensive was identified and outlined in 10 articles. The Lancashire Evening Post (16^th^ May, p32) described the search for GF bread: ‘searching high and low for a brand of GF bread that didn’t taste like cardboard; didn’t fall apart, and didn’t require a second mortgage to afford.’ The cost was identified as a major problem and one of the principal worries when diagnosed with CD. An article by Cambrian News titled ‘Cancer sufferer feared losing her home’ (21^st^ May, Web) outlined a woman under a severe financial strain, struggling to afford GF bread which was over five times more expensive than ‘normal’ bread.

Food content noted as GF

GF food was mentioned and described in 238 articles in a variety of different contexts, which led to the identification of three sub-themes: recipes, products, and eateries. 

## Discussion

This study demonstrates the positive and negative perceptions; and attitudes towards CD and GFDs portrayed in the media, along with the increasing popularity of the GFD. Efforts to raise awareness around the disease and encourage people to seek help - principally by Coeliac UK, as part of their ‘Is it coeliac disease?’ campaign were also documented, as well as the difficulties in getting more people unknowingly living with the condition diagnosed. Additionally, celebrity endorsements were found to play a role in raising awareness and the increasing popularity of GFD. In the three weeks within which the study was conducted, 50% (496) of articles appeared in week one, 25% (245) in week two, and 24% (243) in week three, showing that most coverages occurred the week preceding Coeliac Awareness Week (commencing May 9^th^). 

It was found that articles that negatively portrayed CD or GFDs were most common the week after Awareness Week, with instances during Awareness Week the least common. Furthermore, these articles were found to be most prevalent in national newspapers, giving them an especially wide reach. In particular, the GFD was commonly associated with ‘clean eating’ or ‘healthy eating’, and labeled a ‘fad’ by a handful of newspapers with the negative portrayal of GF products and the cost of GF foods the other key issues identified.

Improving the number of diagnoses of CD was one of the aims of Awareness Week - as part of the larger ‘Is it coeliac disease?’ campaign. Media messaging identified in this study was in line with this, with several articles raising awareness and encouraging people to seek help (if they presented with common symptoms). These articles emphasized an online assessment tool, which checks if further tests are needed to establish a potential diagnosis. Only those with a reasonable chance of CD were recommended to go to their GP for further testing, which may have been a considered way of ensuring no unnecessary strain is placed on GP practices. This online assessment tool is quick and easy to use and therefore is a valuable method for increasing the number of diagnoses by allowing for a larger number of people to be screened. Furthermore, looking at the number of instances by week, it is clear that Awareness Week was a focal point for articles that encouraged people to seek help. Greater exposure to this message in the week preceding Awareness Week increases the importance of the issue in the minds of the public whilst setting up Awareness Week smoothly, as outlined by McCombs’ Agenda Setting Theory [14]. 

Consistent information related to raising awareness and encouragement to seek help, especially by Coeliac UK, allows for the creation of an atmosphere that is conducive to helping people to seek a diagnosis. The online assessment tool is especially useful since it gives people an easier route to check for a potential diagnosis of CD. It relies upon enablement and giving people a ‘nudge’, tying in with Thaler and Sunstein’s Nudge Theory [15]. A nudge is defined as ‘any aspect of choice architecture that alters people’s behavior predictably without forbidding any options or significantly changing their economic incentives’ and is based on indirect encouragement and the design of choices. This method is a good way of increasing diagnosis rates by increasing knowledge and facilitation, which in turn encourages positive decision-making.

Celebrity endorsements were also found to be a channel for consistent messaging and raising awareness for CD. Celebrity endorsement has been defined by McCracken (1989) as ‘Any individual who enjoys public recognition and who uses this recognition on behalf of a consumer good by appearing with it in advertising' [16]. Therefore, the endorsement is important as it builds a congruent image between the brand (in this case, the GFD) and the consumer [17]. Endorsement portrays the GFD in a positive light and therefore encourages those already diagnosed with CD as well as those considering seeking a diagnosis. These celebrity endorsements and articles attempting to raise awareness for CD are notable examples of positive messaging, which are important since positive messages are more effective in influencing health behaviors than negative messages by Richardson et al. [18].

Whilst these messages are likely to empower many people to seek a diagnosis, other messages that portrayed the GFD and GF products negatively may have achieved the opposite. As mentioned previously, negative messaging was found to be more prevalent in week three, perhaps to avoid conflicting with the predominantly positive messages of the mass media campaign or merely ‘drowned out’/replaced by other messages before and during Awareness Week.

Considering the influence of the media, articles that report that GF products could ‘harm the body’ and are ‘not healthy’ may affect the public’s perception of GF products and consequently have a wide range of unintended implications. It has been found that adherence to a GFD is affected by the patient’s perception of the diet [19,20]. Therefore, an increase in negative attitudes and beliefs regarding the GFD among the public may decrease compliance amongst patients with CD, especially those with a recent diagnosis - a worrying effect since a GFD has been found to protect against complications of CD, including osteoporosis, malignancy, and premature mortality, as well as improve quality of life [21-24]. Notions that a GFD is a means to ‘eat clean’ could further affect compliance since patients may find it increasingly difficult to be taken seriously and follow a GFD in the face of rising skepticism. Reluctance to seek a diagnosis may result from negative reporting since the GFD may be dismissed as insignificant by potential patients or labeled as just a ‘fad’, possibly decreasing the rate of diagnosis. Although many articles that are critical of GF products and the GFD make a distinction between following the diet out of medical necessity rather than other reasons, negatively framed articles nonetheless give mixed messages to the public, particularly to those with a potential or recent CD diagnosis, as they contrast with messages given by Coeliac UK’s mass media campaign. This mixed massaging may make it unclear to patients as to whether following a GFD will or will not be significant in managing the disease.

The increased popularity and subsequent growth of the GF industry are reflected by the growing demand for GF products. This is evidenced in the findings of this study by the high number of articles outlining food content noted as GF, especially recipes and products. However, this increased popularity was found to be a double-edged sword. This trend has allowed adhering to a GFD to become easier since GF products are more readily available and commonplace in restaurants/cafes. This has led to an increase in the quality of life for patients with CD by allowing them to lead a more ‘normal’ life. However, much of this increase in demand for GF products could also be attributed to those who follow a GFD without a medical diagnosis, leading some critics to become skeptical of the GFD. This could be a hindrance to those with CD as they struggle to be taken seriously by colleagues, chefs, and even medical professionals, reflecting the results found in other studies [25]. Diagnosis of CD requires the consumption of gluten by suspected patients since gluten restriction can improve histological findings and resolve positive serological test results [26]. Therefore, this increase in self-reported gluten sensitivity and restriction of gluten from the diet by a significant number of individuals may undermine our ability to detect CD. However, in the author’s opinion, the overall benefits associated with the increased popularity of the GFD, such as the increased availability of GF products, outweigh the negatives.

Strengths and limitations

One of the key limitations of this study is that three weeks’ worth of data was analyzed. Therefore, it must be acknowledged that the extent to which the findings can be thought of as representative of the media position and stance of CD and the GFD is limited. The analysis covers Awareness Week, a pivotal week in the calendar year for CD, and so it seems reasonable to suspect media coverage may have been highest at this point. Particularly, it may have also biased the results to be more positive. Moreover, the nature of qualitative research means that findings can be more easily influenced by the researcher’s previous knowledge and personal bias. However, in this study, steps were taken to minimize bias, namely investigator triangulation in relation to the analysis of data, of which 39% was double-coded by the researcher’s supervisors. This further increases the validity and credibility of results, as it ‘gives a more detailed and balanced picture of the situation’ [27]. Additionally, all data were coded by the primary researcher, ensuring consistency of the derived code. 

## Conclusions

In conclusion, it was found that Awareness Week had an influence on the content of newspaper articles since the nature of their content was found to have changed over the three-week period that was analyzed. This was reflected by the significant increase in the number of negatively framed articles in the week after Awareness Week and the significantly higher number of articles in the week preceding Awareness Week. This mixed messaging was considered to negatively impact the perceptions and beliefs of the public, potential patients, and those already diagnosed with CD, regarding CD and the GFD, due to the powerful influence of the media and its ability to set the focus of public attention.

This study has identified the mixed messages given by the media regarding GFD, which may have an impact on the rates of diagnosis of CD as it may deter some from seeking a diagnosis. Therefore, bearing in mind the aims of Coeliac UK’s ‘Is it coeliac disease?’ mass media campaign, it would be beneficial for Coeliac UK to pay particular attention to the GFD. This study recommends that in the future, Coeliac UK focus on and include information regarding the GFD, along with their information, on CD to protect against inaccurate or imprecise information being delivered to potential and current patients. This will help Coeliac UK to achieve its aim of increasing the number of diagnoses by alleviating some of the fears negatively framed articles may instill.
